# Intact Stimulus–Response Conflict Processing in ADHD—Multilevel Evidence and Theoretical Implications

**DOI:** 10.3390/jcm9010234

**Published:** 2020-01-15

**Authors:** Annet Bluschke, Moritz Mückschel, Veit Roessner, Christian Beste

**Affiliations:** Department of Child and Adolescent Psychiatry, Cognitive Neurophysiology, Faculty of Medicine of the TU Dresden, 01309 Dresden, Germany; moritz.mueckschel@ukdd.de (M.M.); veit.roessner@ukdd.de (V.R.); christian.beste@ukdd.de (C.B.)

**Keywords:** attention-deficit-hyperactivity disorder (ADHD), conflict monitoring, EEG, signal decomposition

## Abstract

Attention-deficit-hyperactivity disorder (ADHD) is closely associated with deficits in cognitive control. It seems, however, that the degree of deficits strongly depends on the examined subprocess, with the resolution of stimulus–stimulus conflicts being particularly difficult for patients with ADHD. The picture is far less clear regarding stimulus–response conflicts. The current study provides multi-level behavioural and neurophysiological data on this type of conflict monitoring in children with ADHD compared to healthy controls. To account for the potentially strong effects of intra-individual variability, electroencephalogram (EEG) signal decomposition methods were used to analyze the data. Crucially, none of the analyses (behavioural, event-related potentials, or decomposed EEG data) show any differences between the ADHD group and the control group. Bayes statistical analysis confirmed the high likelihood of the null hypothesis being true in all cases. Thus, the data provide multi-level evidence showing that conflict monitoring processes are indeed partly intact in ADHD, even when eliminating possible biasing factors such as intra-individual variability. While stimulus–stimulus conflict processing has been shown to be consistently dysfunctional in ADHD, the resolution of stimulus–response conflicts is not deficient in this patient group. In comparison to other studies, the results provide novel theoretical insights into the nature of conflict control deficits in childhood ADHD.

## 1. Introduction

Attention-deficit-hyperactivity disorder (ADHD) is one of the most prevalent psychiatric disorders in adolescence and is associated with several deficits in executive functioning and cognitive control [1,2,3,4,5,6,7]. Executive function/cognitive control is an umbrella term for processes which are necessary for goal-directed behaviour [8]. Aside from response inhibition and working memory processes, the control of conflicts and interferences is an important aspect of cognitive control [8].

Overall, the monitoring and control of conflicts depends on an intricate neurophysiological processing architecture associated with the medial and inferior frontal cortices [9,10]. Specifically, conflict monitoring processes are reflected by the N2 [9,11,12]. Crucially, however, studies have suggested that there are different aspects of information being processed during conflict monitoring. For example, perceptual processes are intermingled with processes controlling for incorrect motor response preparation in the medial frontal cortex [9]. This has more recently been corroborated using temporal EEG signal decomposition approaches [13,14,15]. In particular, it has been shown that there are distinct fractions of ‘stimulus codes’ and ‘response selection codes’ in the N2 event-related potential (ERP)-component that are processed in overlapping and adjacent areas of the medial frontal cortex during conflict monitoring [14]. Interestingly, these distinguishable processing codes are associated with distinct neurobiological underpinnings as far as their modulation by the norepinephrine system is concerned [14]. 

The norepinephrine system plays a central role in ADHD and in its treatment [16]. Furthermore, recent data suggest that treatment approaches in ADHD have very specific effects on distinguishable neurophysiological codes associated with cognitive control [17]. All of this already shows that it is necessary to gain more detailed insights into the neurophysiological subprocesses and coding levels underlying different facets of cognitive control in ADHD and to go beyond the commonly examined EEG-parameters for this. This is further underlined by findings showing that there are qualitative differences in how information during cognitive control demands is processed [18,19]. This is particularly important regarding conflict monitoring processes in ADHD, where the evidence is inconsistent [20,21,22,23,24,25] and some evidence suggests that there are no overt changes in conflict-related behavioural adaptations in ADHD [26]. 

It cannot be ruled out that differences in conflict monitoring processes are more subtle in ADHD and can only be delineated when distinct neurophysiological coding levels are isolated in the EEG data. This is all the more likely considering the high intra-individual performance variability in ADHD [27,28,29,30,31], which also affects neurophysiological processes [32,33,34,35,36]. Importantly, commonly used ERP-methods can only yield reliable estimates of neurophysiological processes when the data contains little intra-individual variability [37,38]. It is therefore important to account for this issue using detailed neurophysiological analysis methods (i.e., residue iteration decomposition, RIDE) [37,38].

However, in addition to the above mentioned considerations relating to issues of intra-individual variability and neurophysiological aspects, cognitive-theoretical aspects also have to be considered: At present, the research on conflict monitoring in ADHD is largely inconsistent in relation to theoretical concepts and the different mechanisms underlying conflict monitoring. From a theoretical point of view, stimulus–response (S-R) conflicts have to be distinguished from stimulus–stimulus (S-S) conflicts [39]. An S-S conflict refers to a similarity between stimulus dimensions, whereas an S-R conflict refers to how a relevant stimulus feature is mapped onto a response [39]. The Stroop task (interference effect) is a prominent example of an S-S conflict [40,41], even though S-R processes also play a minor role [39,40,42]. Conflicts evoked by Simon or Flanker tasks are, in contrast, more directly related to S-R conflicts [39]. 

Interestingly, meta-analytic evidence suggests that there are stable conflict monitoring deficits in ADHD when these are examined using S-S conflicts (i.e., the Stroop task) [43]. The pattern is much more diverse and unclear concerning tasks requiring the resolution of S-R conflicts [44]. Notably, S-S conflicts may become resolved by a suppression of irrelevant information [45,46,47] via processes associated with the inferior frontal areas [47]. Intriguingly, (right) inferior frontal regions, involved in inhibitory control processes, have been suggested to reflect the most consistently found abnormality in ADHD [48,49]. This may explain the high consistency of deficits in ADHD as far as Stroop interference effects are concerned [43]. 

Rather different mechanisms and functional neuroanatomical structures seem to be involved in S-R conflicts [50,51]. Specifically, S-R conflicts have consistently been shown to be associated with the medial frontal regions encompassing the anterior cingulate cortex (ACC) and the superior frontal gyrus [11,12,47]. This may explain why patients with ADHD do not exhibit such clear conflict monitoring deficits when S-R conflicts are investigated [44]. In this context, however, it is crucial to control and account for the effects of intra-individual variability. It cannot be excluded that inconsistent findings or the large across-study variation in the severity of deficits can be attributed to intra-individual variability. If, despite controlling for intra-individual variability, no differences to healthy individuals can be observed, this may indicate that there are, in fact, no overarching S-R conflict monitoring deficits in ADHD. This will provide a valuable theoretical constraint to delineate the nature of conflict monitoring deficits in ADHD.

Thus, it is the goal of the current study to examine modulations of neurophysiological processes underlying the resolution of S-R conflicts in ADHD in detail and to account for possible biasing effects of intra-individual variability in the neurophysiological data. Adding to existing studies, the results will provide valuable insights into the nature of conflict monitoring deficits in ADHD. Since a major result of the study could be that there are no deficits in ADHD, the study will also test the evidence for the non-occurrence of an effect/the evidence for the null hypothesis when appropriate. Accordingly, this study uses both classical significance tests and Bayes’ statistics to estimate the degree of evidence for the null hypothesis.

## 2. Experimental Section

### 2.1. Samples and Power Considerations

In total, N = 69 participants were included in the study. All demographic data is presented in Table 1. N = 36 children and adolescents diagnosed with ADHD were included in the study. N = 13 patients were taking ADHD medication (extended release methylphenidate). For comparison, a sample of N = 33 healthy controls was also included in the study. Age, gender, and IQ did not differ significantly between the groups (see Table 1). Thus, these factors were not systematically considered in the further analyses. A sensitivity analysis was computed using the G*Power software package [52]. According to this analysis, and the ANOVA model to analyze the data, the sample is large enough to detect an effect size of f = 0.21 (this equals a ηp^2^ = 0.04) in the important interaction “compatibility x group” with a power of 95%. 

In the ADHD sample, diagnoses had been determined based on standard clinical procedures (i.e., neuropsychological testing of attention, parent and child interviews, teacher reports, symptom questionnaires, the exclusion of potential underlying somatic disorders via EEG, electrocardiogram (ECG), audiometry, and vision testing). Parents rated (0: no problems, 3: severe problems) their children’s ADHD symptoms using the ADHD Symptom Checklist [53]. As expected, patients with ADHD were rated significantly higher in terms of inattention, hyperactivity, and impulsivity (see Table 1). Due to technical reasons, questionnaire data was unfortunately missing from four healthy controls and two patients with ADHD. All subjects and their parents or legal guardians provided informed written consent according to the Declaration of Helsinki. The study was approved by the local ethics committee of the Medical Faculty of the Technical University Dresden (approval code: EK 294092010).

### 2.2. Task

During the flanker task, vertically arranged visual stimuli (white arrowheads) were presented in the centre of a screen on a black background. Central target stimuli pointing to the left or right were preceded (200 ms) by two arrowheads above and below pointing in the same (compatible, 67% of trials) or opposite (incompatible, 33% of trials) direction as the target stimulus. Target stimuli were displayed for 300 ms with flanker stimuli being switched off simultaneously. The response–stimulus interval was pseudo-randomized between 1400 and 1800 ms. To further increase the level of conflict, time pressure was administered since the participants were asked to respond within 450 ms for target onset. If responses occurred after this deadline, a warning tone (1000 Hz, 60 dB sound pressure level) was given 1200 ms after the response. This principal stimulus setup was used in four blocks of 120 stimuli each (i.e., 480 trials in total).

### 2.3. EEG Recording and Analysis

We recorded the EEG signals using an equidistant electrode setup (60 Ag/AgCl electrodes, sampling rate = 500 Hz, reference at Fpz, ground electrode at θ = 58, ф = 78). Impedances were kept below 5 kΩ. Offline data processing took place analogously to the procedure described in (Bluschke et al., 2016): data was down-sampled to 256 Hz and a band-pass filter (0.5–20 Hz, slope: 48 db/oct) was applied. A manual raw data inspection was conducted to remove technical artifacts. Periodically occurring artifacts (pulse artifacts, horizontal, and vertical eye movements) were detected and removed using an independent component analysis. Data was segmented to the onset the target stimulus (−1000–2000 ms). Only trials with correct responses were analysed further. 

An automatic artefact rejection procedure (amplitude criterion: maximal amplitude: +200 µV, minimal amplitude: −200 µV; maximal value difference criterion: 200 μV in a 200 ms interval; and low activity criterion: <0.5 μV in a 100 ms period) was used to remove any remaining artifacts. A current source density transformation was used to allow a reference-free evaluation of the EEG data [54]. Data were then baseline corrected to a time interval from −400 ms to −200 ms before target onset (i.e., interval of 200 ms before flanker onset) and segments were averaged for each condition. 

Using a data-driven approach, single-subject ERP-amplitudes were quantified as the mean amplitude in a defined time interval. The choice of electrodes and time windows was validated using a statistical procedure described in Mückschel et al. [55]. The following electrodes were chosen for ERP quantification: The flanker P1 (−105–−75 ms), flanker N1 (−40–−10 ms), target P1 (110–140 ms), and target N1 (190–220 ms) components were measured over electrodes P7 and P8 (mean). The N2 was quantified over electrode FCz in the time interval of 300–330 ms. The mean amplitude at electrodes CPz and Pz was used to measure the P3 (300–600 ms).

### 2.4. Residue Iteration Decomposition (RIDE)

To account for intra-individual variability in the data, residue iteration decomposition (RIDE) was applied using established protocols [13,14,37,56]. The RIDE toolbox and manual are available at http://cns.hkbu.edu.hk/RIDE.htm. It is important to note that the spatial filter properties of the current source density (CSD) transformation do not violate assumptions of RIDE as the decomposition is conducted separately for each single electrode channel [37]. Full details on the RIDE methods can be found elsewhere [37,38]. RIDE decomposes the ERP single-trials data into three clusters correlated to the stimulus onset (S-cluster) or to the response time (R-cluster). Further, a third intermediate cluster (C-cluster) with variable latency is identified, which is estimated initially and iteratively improved. RIDE uses a nested iteration scheme for latency estimation through which the latency estimation of the C-cluster is improved. The initial latency of the C-cluster is estimated using a time window function. 

Subsequently, the S-cluster is removed and the latency of the C-cluster is re-estimated based on a template matching approach. Information about the validity of the template matching approach used by the RIDE algorithm can be found elsewhere [37,38,57]. During processing, the initial time window for the estimation of the C-cluster was set at 200 to 900 ms after stimulus onset. The time window is assumed to cover the range within which each component is supposed to occur [37]. The time window for the S-cluster was set at −400 to 600 ms around target onset. For the R-cluster, the time window was set at −300 to 300 ms around the response. 

The choice of electrodes and time windows to quantify the RIDE clusters were also validated using a statistical procedure described in Mückschel et al. [55]. For the RIDE-based analysis, the following electrodes were chosen on the basis of the scalp topography: In the S-cluster, the flankerP1RIDE (−110 to −80 ms), the flankerN1RIDE (−40 to −10 ms), the targetP1RIDE (110–140 ms), and the targetN1RIDE (190–220 ms) were measured over electrodes P7 and P8 (mean). The S-cluster N2 RIDE component (300–330 ms) was quantified at electrode Fz. 

The C-cluster data was used to quantify activation in the N2 and P3 time windows. The C-cluster N2 RIDE component (compatible: 415–445 ms, incompatible: 535–565 ms) was quantified at electrode Fz. The P3 RIDE was measured in the time window of 300–600 ms at electrode Pz. In the R-Cluster, relevant activation was quantified in a time interval of ±100 ms around the mean reaction time (compatible trials: 232–432 ms, incompatible trials: 316–516 ms) at electrodes C3 and C4 (mean).

### 2.5. Statistics

In all analyses, means and standard errors are indicated as descriptive statistics. Pearson correlations were calculated in order to examine any associations between ADHD symptom severity and the other variables. Reaction times (RTs) in corrects trials were analyzed using repeated-measures ANOVA, using the within-subject factor Compatibility (compatible vs. incompatible) and the between-subject factor Group (controls vs. ADHD). The same statistical model was used to analyse the error rates (% of errors) as well as the neurophysiological data. The effect of Compatibility was not analysed for any flanker-related neurophysiological data, since this effect can only occur after presentation of a target stimulus. Greenhouse–Geisser correction was applied and post-hoc tests, as well as all correlational analyses, were Bonferroni-corrected when necessary. To substantiate the lack of significant effects, we performed Bayesian analysis [58] to calculate the probability of the null hypothesis being true given the obtained data *p*(H0/D). The entire dataset can be obtained from the Supplementary Materials.

## 3. Results

### 3.1. Behavioural Data

Concerning reaction times (RTs), there were no significant differences (F (1,67) = 0.01; *p* = 0.92; ηp^2^ = 0.0) between the groups (controls: 373 ± 12.5 ms; ADHD: 374 ± 11.9 ms). The repeated-measures ANOVA further showed significantly faster reaction times in compatible (331 ± 7.9 ms) than in incompatible (416 ± 10.0 ms) trials (main effect Compatibility: F (1, 67) = 238.1; *p* ≤ 0.001; ηp^2^ = 0.78). The interaction of Group * Compatibility was not significant (F (1, 67) = 0.05; *p* = 0.82; ηp^2^ = 0.001), with Bayesian analysis showing *p*(H0/D) = 0.89. According to Raftery [59], this shows that there is strong evidence in favor of the null hypothesis. A similar pattern emerged concerning error rates. Here, there were also no significant differences (F (1,67) = 1.6; *p* = 0.21; ηp^2^ = 0.02) between groups (controls: 32.6 ± 2.2%; ADHD: 28.5 ± 2.3%). Both groups committed significantly more errors in incompatible (44.0 ± 1.9%) than in compatible trials (17.1 ± 2.2%) (F (1, 67) = 111.2; *p* ≤ 0.001; ηp^2^ = 0.62). The interaction of Group * Compatibility was not significant (F (1, 67) = 0.55; *p* = 0.46; ηp^2^ = 0.008). Bayesian analysis revealed *p*(H0/D) = 0.86. Further, ADHD symptom severity was not correlated with task performance (all r ≤ 0.24, all *p* ≥ 0.057).

### 3.2. Neurophysiological Data

#### 3.2.1. Standard ERP Analysis

**Figure 1:**
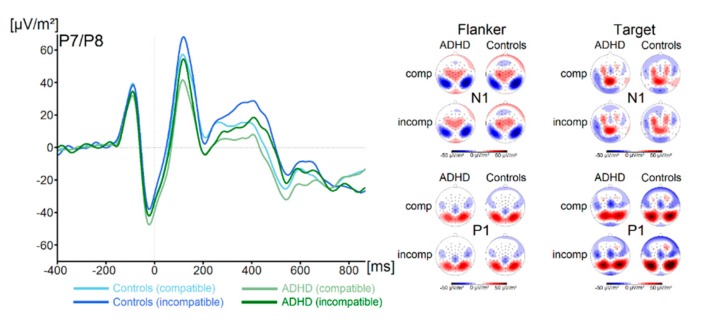
The P1 and N1 ERP-components are shown pooled across electrodes P7 and P8. Time point zero denotes the time point of target stimulus presentation. Negativity is plotted downwards. The different colours of the ERP traces denote the compatible (lighter tone) and incompatible (darker tone) trials in patients with ADHD (green) and controls (blue). The scalp topography plots are shown for the peak of each ERP component in the compatibility conditions and groups. In the maps, blue colours denote negativity and red colours denote positivity.

P1 and N1 components in response to the flanker and the target stimuli for both groups and both trial types are shown in Figure 1.

**Figure 2:**
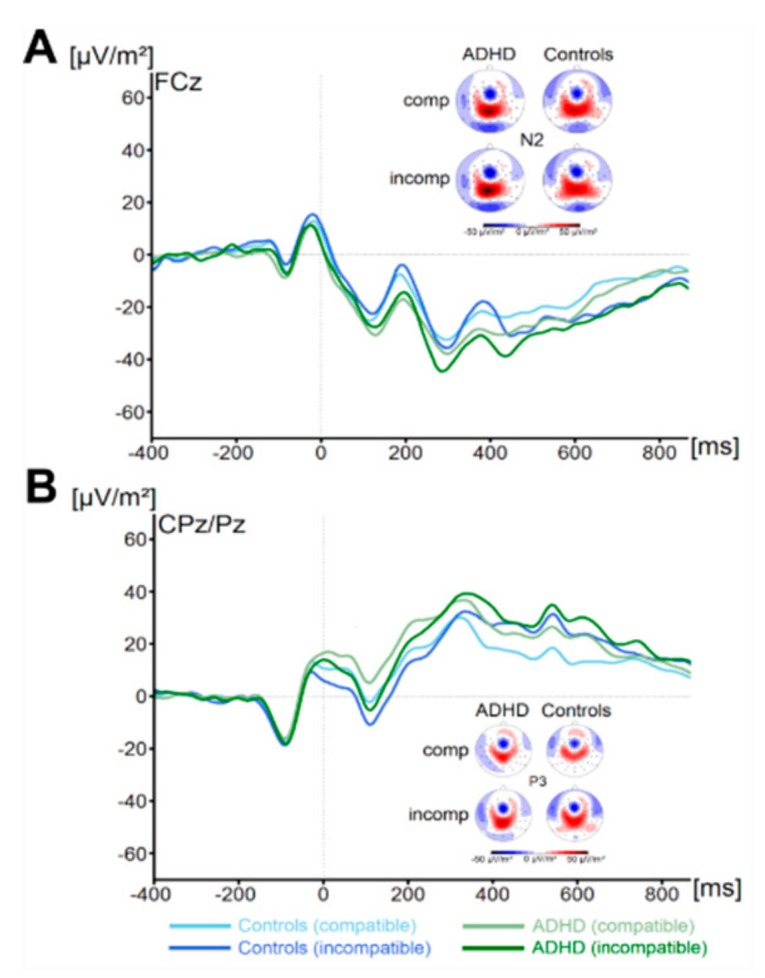
The N2 (Figure part **A**) and P3 ERP-component (Figure part **B**) are shown at electrodes FCz and pooled across CPz and Pz, respectively. Time point zero denotes the time point of target stimulus presentation. Negativity is plotted downwards. The different colours of the ERP traces denote the compatible (lighter tone) and incompatible (darker tone) trials in patients with ADHD (green) and controls (blue). The scalp topography plots are shown for the peak of each ERP component in the compatibility conditions and groups. In the maps, blue colours denote negativity and red colours denote positivity.

There were no significant main effects of the factor Group on flanker P1 or N1 amplitude (all F < 1.1, all *p* > 0.29, all ηp^2^ < 0.02). Concerning the target P1, we found a main effect of Compatibility (F (1,67) = 74.4, *p* ≤ 0.001, ηp^2^ = 0.5) There were no further significant main effects or interactions concerning target P1 amplitude (all F < 3.1, all *p* > 0.08, all ηp^2^ < 0.04). Concerning the target N1 amplitude, we found no significant main effects or interactions (all F < 1.3, all *p* > 0.26, all ηp^2^ < 0.02). Concerning the N2 (see Figure 2), we also found no main effects or interactions (all F < 1.6, all *p* > 0.21, all ηp^2^ < 0.02). Analysis of P3 amplitude (see Figure 2) revealed a main effect of Compatibility (F (1,67) = 20.2, *p* ≤ 0.001, ηp^2^ = 0.23). All other effects were not significant (all F < 1.0, all *p* > 0.31, all ηp^2^ < 0.015). Using Bayesian analyses, we could show for the probability of the null hypothesis being true for all six non-significant Group * Compatibility to be *p*(H0/D) ≥ 0.78. ADHD symptom severity was not correlated with any of these measures (all r ≤ 0.26, all *p* ≥ 0.04).

#### 3.2.2. RIDE Analysis

##### S-Cluster

**Figure 3:**
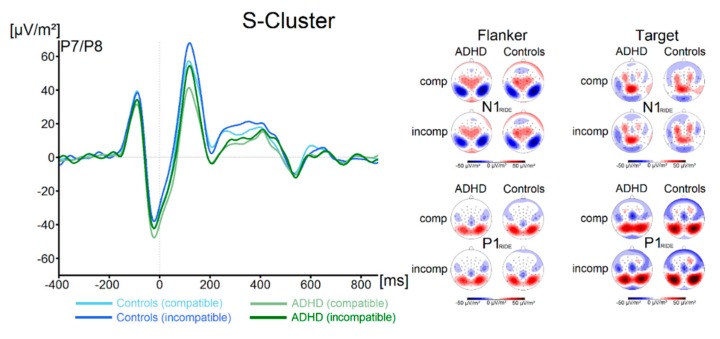
The flanker and target P1 _RIDE_ and N1 _RIDE_ ERPs in the S-cluster are shown pooled across electrodes P7 and P8. Time point zero denotes the time point of target stimulus presentation. Negativity is plotted downwards. The different colours of the ERP traces denote the compatible (lighter tone) and incompatible (darker tone) trials in patients with ADHD (green) and controls (blue). The scalp topography plots are shown for the peak of each ERP component in the compatibility conditions and groups. In the maps, blue colours denote negativity and red colours denote positivity.

Components in the flanker P1-time window (flanker P1_RIDE_), flanker N1-time window (flanker N1_RIDE_), the target P1-time window (target P1_RIDE_) and in the target N1-time window (target N1_RIDE_) for both groups and both trial types are shown in Figure 3.

**Figure 4:**
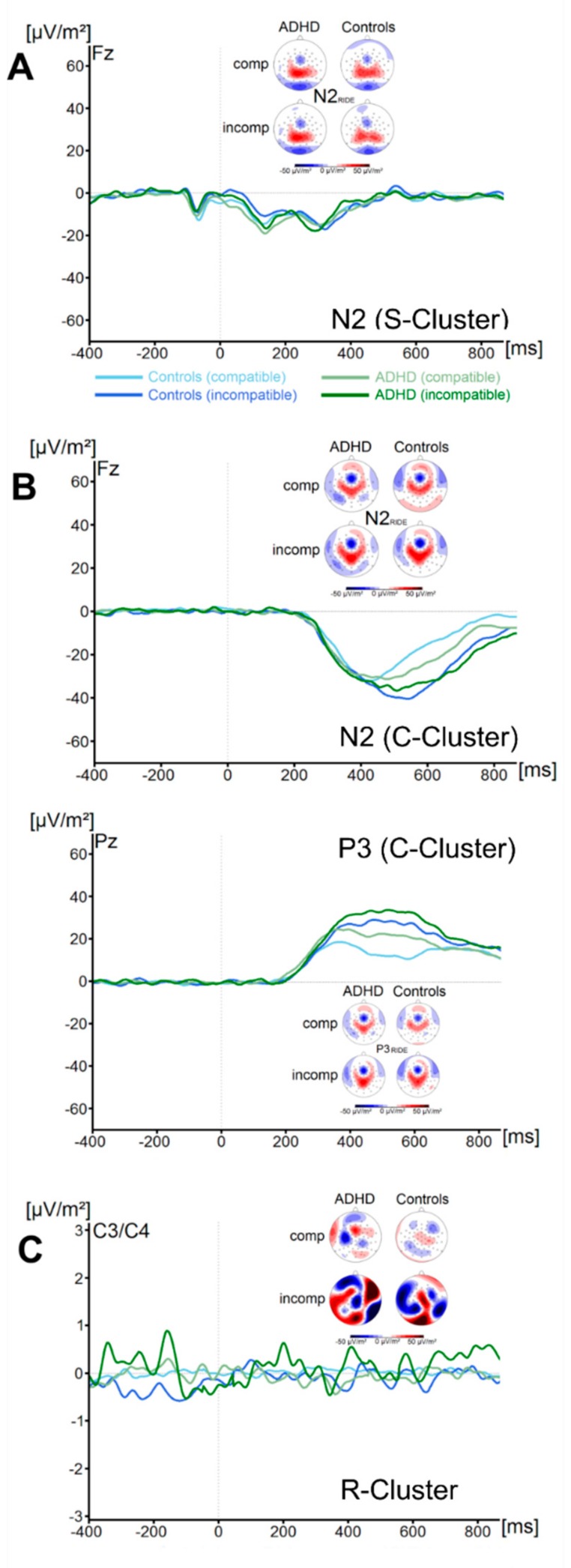
(**A**) The S-cluster in the N2 _RIDE_ time window is shown at electrode Fz. (**B**) The C-cluster in the N2 _RIDE_ time window is shown at electrode Fz (top) and the C-cluster in the P3 _RIDE_ time window at electrode Pz (bottom). (**C**) The R-cluster is shown pooled across electrodes C3 and C4. Time point zero denotes the time point of target stimulus presentation. Negativity is plotted downwards. The different colours of the ERP traces denote the compatible (lighter tone) and incompatible (darker tone) trials in patients with ADHD (green) and controls (blue). The scalp topography plots are shown for the peak of each ERP component in the compatibility conditions and groups. In the maps, blue colours denote negativity and red colours denote positivity.

There were no significant effects of the factor Group on the flanker P1_RIDE_ amplitude (all F < 2.1, all *p* > 0.16, all ηp^2^ < 0.03). Concerning the flanker N1_RIDE_ amplitude, we also found no significant main effects or interactions (all F < 0.58, all *p* > 0.45, all ηp^2^ < 0.009). Analysis of the target P1_RIDE_ amplitude revealed a significant main effect of Compatibility (F (1, 67) = 73.9; *p* ≤ 0.001; ηp^2^ = 0.52), with incompatible trials resulting in significantly higher amplitudes (59.3 ± 5.0 µV/m^2^) than compatible trials (47.5 ± 4.6 µV/m^2^). All other main effects and interactions were not significant (all F < 3.1, all *p* > 0.08, all ηp^2^ < 0.04). No significant main effects or interactions were found for the target N1_RIDE_ amplitude (all F < 1.1, all *p* > 0.29, all ηp^2^ < 0.02). Using Bayesian analyses, we could show for the probability of the null hypothesis being true for all four non-significant Group * Compatibility to be *p*(H0/D) ≥ 0.72. The waveform of the S-cluster in the N2-time window (N2 _RIDE_S_) for both groups and for compatible and incompatible trials is shown in Figure 4A.

The analyses of the N2_RIDE_S_ amplitude revealed no main effects or interactions (all F < 0.5, all *p* > 0.49, all ηp^2^ < 0.007) were significant. Bayesian analyses showed for the probability of the null hypothesis being true for the non-significant Group * Compatibility to be *p*(H0/D) = 0.89. ADHD symptom severity was not correlated with any of these measures (all r ≤ −0.2, all *p* ≥ 0.12).

##### C-Cluster

The waveform of the C-cluster in the N2-time window (N2_RIDE_C_) for both groups and for compatible and incompatible trials is shown in Figure 4B. The analyses of the N2 _RIDE_C_ component revealed a main effect of Compatibility (F (1, 67) = 6.5; *p* = 0.01; ηp^2^ = 0.09). Incompatible trials (−37.2 ± 3.5 µV/m^2^) resulted in significantly more pronounced N2 _RIDE_C_ amplitudes than compatible trials (−31.1 ± 2.6 µV/m^2^). All other main effects and interactions were not significant (all F < 0.9, all *p* > 0.35, all ηp^2^ < 0.013). Bayesian analyses showed for the probability of the null hypothesis being true for the non-significant Group * Compatibility to be p(H0/D) = 0.83. The waveform of the C-cluster in the P3-time window (P3_RIDE_) at electrode Pz for both groups and for compatible and incompatible trials is shown in Figure 4B. The analyses of the P3_RIDE_ component revealed a main effect of Compatibility (F (1, 67) = 29.8; *p* ≤ 0.001; ηp^2^ = 0.3). Incompatible trials (27.4 ± 2.9 µV/m^2^) resulted in significantly more pronounced P3_RIDE_ amplitudes than compatible trials (17.4 ± 2.1 µV/m^2^). All other main effects and interactions were not significant (all F < 0.72, all *p* > 0.4, all ηp^2^ < 0.01). Bayesian analyses showed for the probability of the null hypothesis being true for the non-significant Group * Compatibility to be p(H0/D) = 0.86. ADHD symptom severity was not correlated with any of these measures (all r ≤ 0.26, all *p* ≥ 0.04).

##### R-Cluster

The waveform of the R-Cluster for both groups and for compatible and incompatible trials is shown in Figure 4C. Analyses revealed no significant main effects or interactions (all F < 0.7, all *p* > 0.41, all ηp^2^ < 0.010). Bayesian analyses showed for the probability of the null hypothesis being true for the non-significant Group * Compatibility to be p(H0/D) = 0.85. ADHD symptom severity was not correlated with R-Cluster activity (all r ≤ −0.25, all *p* ≥ 0.05).

## 4. Discussion

In the current study, we examined conflict monitoring processes in patients with ADHD using a Flanker task and neurophysiological methods. Dysfunctions in various cognitive control functions such as conflict processing are supposed to reflect a major aspect of ADHD [1,2,3,4,5,6,7]. The current study was motivated by the fact that current knowledge on this important element of cognitive control in ADHD may be biased by intra-individual variability, known to be high in this population [27,28,29,30,31]. This variability may partly explain the inconsistent findings in conflict monitoring in ADHD. In particular, this could be the case concerning neurophysiological correlates, since these can only yield reliable estimates of neurophysiological processes when there is little intra-individual variability in the data [37,38]. In addition, research on conflict monitoring in ADHD has so far often disregarded different mechanisms and theoretical concepts that could play a role and may be differently affected in ADHD.

The results show that ADHD is not associated with deficits in conflict monitoring when examined using a Flanker task. The study is well powered (cf. sensitivity analysis) and the lack of group differences is substantiated with a Bayesian analysis of the data. The latter provided positive evidence in favour for the null hypothesis, i.e., that there are no deficits in conflict monitoring in ADHD. This was the case for the behavioural and the neurophysiological data. Notably, we found strong evidence for the null hypothesis even when accounting for intra-individual variability in the data using RIDE, and when considering that different aspects of information processing during conflict monitoring are coded in distinct parts of the neurophysiological signal. Further, ADHD symptom severity was not correlated with any of these parameters. Thus, the data provide multi-level behavioural and neurophysiological evidence showing that conflict monitoring processes are indeed partly intact in ADHD. Importantly, when looking closer at the available evidence on conflict monitoring in ADHD in comparison to the current results, the nature of cognitive control deficits in ADHD becomes clearer:

As mentioned above, previous evidence on conflict monitoring processes in ADHD is heterogenous [27,28,29,30,31]. Mullane et al. [44] compared studies on conflict monitoring processes in ADHD using Flanker tasks—the type of task that was also used in the current study. The authors showed that observed congruency effects strongly differed between studies. The current findings also corroborate that no robust differences between children with ADHD and healthy controls are evident. However, a meta-analysis on studies using another paradigm to examine conflict monitoring (i.e., the Stroop task) concluded that conflict monitoring is consistently dysfunctional in ADHD [43]. 

This apparent variation in conflict monitoring deficits depending on the approach (i.e., test/task) used to assess conflict monitoring provides insights into the boundary conditions under which conflict/interference monitoring is dysfunctional in ADHD. Response interference, as examined in the Flanker task or Simon task [44], involves competition between two task-relevant responses [60]. From a theoretical perspective, conflicts in these tasks have been framed as stimulus–response (S-R) conflicts [40], which have to be distinguished from stimulus–stimulus (S-S) conflicts [39]. The S-S conflict refers to a similarity between stimulus dimensions, whereas the S-R conflict refers to how a relevant stimulus feature is mapped on a response [39]. S-S conflicts strongly and independently contribute to the Stroop interference effect [42,61,62]. 

From this cognitive-theoretical perspective, it seems that conflict monitoring is consistently dysfunctional in ADHD when it comes to the resolution of S-S conflicts (i.e., Stroop effects [43]), but not when it comes to the resolution of S-R conflicts (i.e., Flanker effects). The multi-level behavioural and neurophysiological data provided in this study is in line with that. Importantly, Stroop (S-S) and Simon/Flanker (S-R) inference effects represent distinct entities, with S-R and S-S conflicts also being processed at different times [50,51]. It has been shown that S-S conflicts do not strongly modulate processes in earlier (i.e., N2) time windows, while this was the case for S-R conflicts [50,51]. Likely, S-S conflicts require time-consuming suppression of irrelevant information [45,46,47], while S-R conflicts do not depend on “inhibitory control” mechanisms as strongly [50,51]. 

Notably, meta-analytic functional imaging data show that especially S-S conflicts modulate processes in the inferior frontal and anterior cingulate areas [47], i.e., areas that are part of a cortical response inhibition network [63,64]. EEG source localization data provides evidence that (inferior) prefrontal structures and inhibitory control processes are involved during the resolution of S-S conflicts [50,65,66]. Intriguingly, (right) inferior frontal regions, involved in inhibitory control processes, have been suggested to reflect the most consistently found differences in ADHD [48,49]. 

Overall, it has further been argued that deficits in ‘inhibitory control’ are at the core of cognitive control deficits in ADHD and carry a high clinical relevance [67,68,69,70,71,72]. Taking this perspective, the importance of inhibitory control processes for conflict processing may explain why consistent ADHD-related deficits have been observed in S-S conflicts [43], but not in other sorts of conflicts (as in the current study). It is possible that deficits in conflict monitoring in ADHD only become apparent when a strong contribution of inhibitory control is necessary to resolve the conflict. 

From a clinical perspective it seems that it is important to strengthen inhibitory control processes in ADHD. This is especially important as baseline impulsivity has been suggested to be a very good predictor of treatment response [73]. Interestingly, treatment approaches like neurofeedback have been shown to lead to improvements in inhibitory control and to decreases in impulsivity in patients with ADHD [74,75]. From the point of view of medication, it has been suggested that atomoxetine (frequently used as second-line medication) may be more effective in reducing impulsivity than is the case for methylphenidate (first-line treatment), which may in turn be more beneficial in terms of reducing inattention [76,77]. 

Importantly, only atomoxetine has been shown to increase prefrontal activation during a Stroop task [78]. This effect may however crucially depend on different demographic factors and on individual differences in symptom severity [78]. Taken together, this suggests that it may indeed be very useful to take the individual pattern of cognitive deficits into account when developing treatment recommendations for individual patients [79]. Specifically, patients with higher impulsivity scores and more problems in S-S conflict resolution may benefit more from atomoxetine than methylphenidate (MPH) treatment. 

Future studies should therefore evaluate whether there are differences in the treatment effects depending on whether S-R or S-S conflicts are examined in ADHD and whether such results should be included in the calculation of risk scores or information profiles, which might guide treatment decisions in the future [80]. Importantly, such approaches could also take pharmacogenetic approaches into account, as genetic factors likely underlie the large interindividual variation in the response to different pharmacological treatments [81].

## Figures and Tables

**Table 1:** Sample description. Attention-deficit-hyperactivity disorder (ADHD). The mean and standard error of the mean (SE) are given.

	Healthy Controls (*n* = 33)	Patients with ADHD (*n* = 36)	Group Comparison
***n* male**	21	24	*Χ*^2^(1) = 0.07, *p* = 0.79
**mean age ± SE**	11.7 ± 0.4 years	11.7 ± 0.3 years	*t*(68) = 0.11; *p* = 0.92
**mean IQ ± SE**	106 ± 1.3	102 ± 1.7	*t*(68) = −1.8; *p* = 0.08
**ADHD symptom checklist inattention (mean ± SE)**	0.5 ± 0.1	2.0 ± 0.1	*t*(62) = 11.2; *p* ≤ 0.001
**ADHD symptom checklist hyperactivity (mean ± SE)**	0.09 ± 0.05	1.1 ± 0.1	*t*(62) = 7.9; *p* ≤ 0.001
**ADHD symptom checklist impulsivity (mean ± SE)**	0.3 ± 0.09	1.5 ± 0.2	*t*(62) = 6.5; *p* ≤ 0.001

## Supplementary Materials

The data structure containing all analysed data can be found at https://osf.io/4zgue/?view_only=2c31a3f1f5534668aaab557377bdf4ee.

## References

[1] Ahmadi N., Mohammadi M.R., Araghi S.M., Zarafshan H. (2014). Neurocognitive profile of children with attention deficit hyperactivity disorders (ADHD): A comparison between subtypes. Iran. J. Psychiatry.

[2] Arnsten A.F.T., Rubia K. (2012). Neurobiological circuits regulating attention, cognitive control, motivation, and emotion: Disruptions in neurodevelopmental psychiatric disorders. J. Am. Acad. Child Adolesc. Psychiatry.

[3] Oosterlaan J., Sergeant J.A. (1998). Response inhibition and response re-engagement in attention-deficit/hyperactivity disorder, disruptive, anxious and normal children. Behav. Brain Res..

[4] Randall K.D., Brocki K.C., Kerns K.A. (2009). Cognitive control in children with ADHD-C: How efficient are they?. Child Neuropsychol. J. Norm. Abnorm. Dev. Child. Adolesc..

[5] Stroux D., Shushakova A., Geburek-Höfer A.J., Ohrmann P., Rist F., Pedersen A. (2016). Deficient interference control during working memory updating in adults with ADHD: An event-related potential study. Clin. Neurophysiol. Off. J. Int. Fed. Clin. Neurophysiol..

[6] Urcelay G.P., Dalley J.W. (2012). Linking ADHD, impulsivity, and drug abuse: A neuropsychological perspective. Curr. Top. Behav. Neurosci..

[7] Van Rooij D., Hartman C.A., Mennes M., Oosterlaan J., Franke B., Rommelse N., Heslenfeld D., Faraone S.V., Buitelaar J.K., Hoekstra P.J. (2015). Altered neural connectivity during response inhibition in adolescents with attention-deficit/hyperactivity disorder and their unaffected siblings. NeuroImage Clin..

[8] Diamond A. (2013). Executive functions. Annu. Rev. Psychol..

[9] Folstein J.R., Van Petten C. (2008). Influence of cognitive control and mismatch on the N2 component of the ERP: A review. Psychophysiology.

[10] Shenhav A., Botvinick M.M., Cohen J.D. (2013). The expected value of control: An integrative theory of anterior cingulate cortex function. Neuron.

[11] Carter C.S., van Veen V. (2007). Anterior cingulate cortex and conflict detection: An update of theory and data. Cogn. Affect. Behav. Neurosci..

[12] Larson M.J., Clayson P.E., Clawson A. (2014). Making sense of all the conflict: A theoretical review and critique of conflict-related ERPs. Int. J. Psychophysiol. Off. J. Int. Organ. Psychophysiol..

[13] Chmielewski W.X., Mückschel M., Beste C. (2018). Response selection codes in neurophysiological data predict conjoint effects of controlled and automatic processes during response inhibition. Hum. Brain Mapp..

[14] Mückschel M., Chmielewski W., Ziemssen T., Beste C. (2017). The norepinephrine system shows information-content specific properties during cognitive control—Evidence from EEG and pupillary responses. NeuroImage.

[15] Mückschel M., Dippel G., Beste C. (2017). Distinguishing stimulus and response codes in theta oscillations in prefrontal areas during inhibitory control of automated responses. Hum. Brain Mapp..

[16] Faraone S.V. (2018). The pharmacology of amphetamine and methylphenidate: Relevance to the neurobiology of attention-deficit/hyperactivity disorder and other psychiatric comorbidities. Neurosci. Biobehav. Rev..

[17] Bluschke A., Friedrich J., Schreiter M.L., Roessner V., Beste C. (2018). A comparative study on the neurophysiological mechanisms underlying effects of methylphenidate and neurofeedback on inhibitory control in attention deficit hyperactivity disorder. NeuroImage Clin..

[18] Bluschke A., von der Hagen M., Papenhagen K., Roessner V., Beste C. (2017). Response inhibition in attention deficit disorder and neurofibromatosis type 1-clinically similar, neurophysiologically different. Sci. Rep..

[19] Chmielewski W., Bluschke A., Bodmer B., Wolff N., Roessner V., Beste C. (2019). Evidence for an altered architecture and a hierarchical modulation of inhibitory control processes in ADHD. Dev. Cogn. Neurosci..

[20] Albrecht B., Brandeis D., Uebel H., Heinrich H., Mueller U.C., Hasselhorn M., Steinhausen H.-C., Rothenberger A., Banaschewski T. (2008). Action monitoring in boys with attention-deficit/hyperactivity disorder, their nonaffected siblings, and normal control subjects: Evidence for an endophenotype. Biol. Psychiatry.

[21] Dockstader C., Gaetz W., Cheyne D., Tannock R. (2009). Abnormal neural reactivity to unpredictable sensory events in attention-deficit/hyperactivity disorder. Biol. Psychiatry.

[22] Karatekin C., Bingham C., White T. (2009). Regulation of cognitive resources during an n-back task in youth-onset psychosis and attention-deficit/hyperactivity disorder (ADHD). Int. J. Psychophysiol..

[23] Sergeant J.A., van der Meere J. (1988). What happens after a hyperactive child commits an error?. Psychiatry Res..

[24] Spinelli S., Joel S., Nelson T.E., Vasa R.A., Pekar J.J., Mostofsky S.H. (2011). Different neural patterns are associated with trials preceding inhibitory errors in children with and without attention-deficit/hyperactivity disorder. J. Am. Acad. Child Adolesc. Psychiatry.

[25] Van Meel C.S., Heslenfeld D.J., Oosterlaan J., Sergeant J.A. (2007). Adaptive control deficits in attention-deficit/hyperactivity disorder (ADHD): The role of error processing. Psychiatry Res..

[26] Bluschke A., Chmielewski W.X., Roessner V., Beste C. Intact context-dependent modulation of conflict monitoring in childhood ADHD. J. Atten. Disord..

[27] Gmehlin D., Fuermaier A.B.M., Walther S., Debelak R., Rentrop M., Westermann C., Sharma A., Tucha L., Koerts J., Tucha O. (2014). Intraindividual variability in inhibitory function in adults with ADHD—An ex-Gaussian approach. PLoS ONE.

[28] Henríquez-Henríquez M.P., Billeke P., Henríquez H., Zamorano F.J., Rothhammer F., Aboitiz F. (2014). Intra-individual response variability assessed by ex-gaussian analysis may be a new endophenotype for attention-deficit/hyperactivity disorder. Front. Psychiatry.

[29] Lin H.-Y., Hwang-Gu S.-L., Gau S.S.-F. (2015). Intra-individual reaction time variability based on ex-Gaussian distribution as a potential endophenotype for attention-deficit/hyperactivity disorder. Acta Psychiatr. Scand..

[30] Plessen K.J., Allen E.A., Eichele H., van Wageningen H., Høvik M.F., Sørensen L., Worren M.K., Hugdahl K., Eichele T. (2016). Reduced error signalling in medication-naive children with ADHD: Associations with behavioural variability and post-error adaptations. J. Psychiatry Neurosci..

[31] Saville C.W.N., Feige B., Kluckert C., Bender S., Biscaldi M., Berger A., Fleischhaker C., Henighausen K., Klein C. (2015). Increased reaction time variability in attention-deficit hyperactivity disorder as a response-related phenomenon: Evidence from single-trial event-related potentials. J. Child Psychol. Psychiatry.

[32] Alba G., Pereda E., Mañas S., Méndez L.D., Duque M.R., González A., González J.J. (2016). The variability of EEG functional connectivity of young ADHD subjects in different resting states. Clin. Neurophysiol. Off. J. Int. Fed. Clin. Neurophysiol..

[33] Bluschke A., Gohil K., Petzold M., Roessner V., Beste C. (2018). Neural mechanisms underlying successful and deficient multi-component behavior in early adolescent ADHD. NeuroImage Clin..

[34] Bluschke A., Chmielewski W.X., Mückschel M., Roessner V., Beste C. (2017). Neuronal intra-individual variability masks response selection differences between ADHD subtypes—A need to change perspectives. Front. Hum. Neurosci..

[35] Gonen-Yaacovi G., Arazi A., Shahar N., Karmon A., Haar S., Meiran N., Dinstein I. (2016). Increased ongoing neural variability in ADHD. Cortex J. Devoted Study Nerv. Syst. Behav..

[36] Lazzaro I., Anderson J., Gordon E., Clarke S., Leong J., Meares R. (1997). Single trial variability within the P300 (250–500 ms) processing window in adolescents with attention deficit hyperactivity disorder. Psychiatry Res..

[37] Ouyang G., Sommer W., Zhou C. (2015). A toolbox for residue iteration decomposition (RIDE)—A method for the decomposition, reconstruction, and single trial analysis of event related potentials. J. Neurosci. Methods.

[38] Ouyang G., Herzmann G., Zhou C., Sommer W. (2011). Residue iteration decomposition (RIDE): A new method to separate ERP components on the basis of latency variability in single trials. Psychophysiology.

[39] Hasshim N., Parris B.A. (2015). Assessing stimulus–stimulus (semantic) conflict in the Stroop task using saccadic two-to-one color response mapping and preresponse pupillary measures. Atten. Percept. Psychophys..

[40] Hommel B. (2011). The Simon effect as tool and heuristic. Acta Psychol. (Amst).

[41] Kornblum S., Hasbroucq T., Osman A. (1990). Dimensional overlap: Cognitive basis for stimulus-response compatibility—A model and taxonomy. Psychol. Rev..

[42] De Houwer J. (2003). On the role of stimulus-response and stimulus-stimulus compatibility in the Stroop effect. Mem. Cognit..

[43] Lansbergen M.M., Kenemans J.L., van Engeland H. (2007). Stroop interference and attention-deficit/hyperactivity disorder: A review and meta-analysis. Neuropsychology.

[44] Mullane J.C., Corkum P.V., Klein R.M., McLaughlin E. (2009). Interference control in children with and without ADHD: A systematic review of Flanker and Simon task performance. Child Neuropsychol. J. Norm. Abnorm. Dev. Child. Adolesc..

[45] Coderre E.L., van Heuven W.J.B. (2013). Modulations of the executive control network by stimulus onset asynchrony in a Stroop task. BMC Neurosci..

[46] Lei H., Yi J., Wang H., Zhang X., Dong J., Zhou C., Fan J., Zhong M., Zhu X. (2013). Inhibitory deficit in semantic conflict in obsessive-compulsive disorder: An event-related potential study. Neurosci. Lett..

[47] Li Q., Yang G., Li Z., Qi Y., Cole M.W., Liu X. (2017). Conflict detection and resolution rely on a combination of common and distinct cognitive control networks. Neurosci. Biobehav. Rev..

[48] Hart H., Radua J., Nakao T., Mataix-Cols D., Rubia K. (2013). Meta-analysis of functional magnetic resonance imaging studies of inhibition and attention in attention-deficit/hyperactivity disorder: Exploring task-specific, stimulant medication, and age effects. JAMA Psychiatry.

[49] Rubia K., Alegria A.A., Cubillo A.I., Smith A.B., Brammer M.J., Radua J. (2014). Effects of stimulants on brain function in attention-deficit/hyperactivity disorder: A systematic review and meta-analysis. Biol. Psychiatry.

[50] Chmielewski W.X., Beste C. (2019). Stimulus feature conflicts enhance motor inhibitory control processes in the lateral prefrontal Cortex. J. Cogn. Neurosci..

[51] Chmielewski W.X., Beste C. (2019). Neurophysiological mechanisms underlying the modulation of cognitive control by simultaneous conflicts. Cortex J. Devoted Study Nerv. Syst. Behav..

[52] Faul F., Erdfelder E., Lang A.-G., Buchner A.G. (2007). Power 3: A flexible statistical power analysis program for the social, behavioral, and biomedical sciences. Behav. Res. Methods.

[53] Döpfner M., Görtz-Dorten A., Lehmkuhl G. (2008). Diagnostik-System für Psychische Störungen im Kindes- und Jugendalter nach ICD-10 und DSM-IV, DISYPS-II.

[54] Nunez P.L., Pilgreen K.L. (1991). The spline-Laplacian in clinical neurophysiology: A method to improve EEG spatial resolution. J. Clin. Neurophysiol. Off. Publ. Am. Electroencephalogr. Soc..

[55] Mückschel M., Stock A.-K., Beste C. (2014). Psychophysiological mechanisms of interindividual differences in goal activation modes during action cascading. Cereb. Cortex.

[56] Ouyang G., Sommer W., Zhou C. (2015). Updating and validating a new framework for restoring and analyzing latency-variable ERP components from single trials with residue iteration decomposition (RIDE). Psychophysiology.

[57] Ouyang G., Schacht A., Zhou C., Sommer W. (2013). Overcoming limitations of the ERP method with Residue Iteration Decomposition (RIDE): A demonstration in go/no-go experiments. Psychophysiology.

[58] Masson M.E.J. (2011). A tutorial on a practical Bayesian alternative to null-hypothesis significance testing. Behav. Res. Methods.

[59] Raftery A.E. (1995). Bayesian model selection in social research. Sociol. Methodol..

[60] Sebastian A., Jung P., Krause-Utz A., Lieb K., Schmahl C., Tüscher O. (2014). Frontal dysfunctions of impulse control—A systematic review in borderline personality disorder and attention-deficit/hyperactivity disorder. Front. Hum. Neurosci..

[61] Zhang H., Kornblum S. (1998). The effects of stimulus–response mapping and irrelevant stimulus–response and stimulus–stimulus overlap in four-choice Stroop tasks with single-carrier stimuli. J. Exp. Psychol. Hum. Percept. Perform..

[62] Schmidt J.R., Cheesman J. (2005). Dissociating stimulus-stimulus and response-response effects in the stroop task. Can. J. Exp. Psychol. Can. Psychol. Expérimentale.

[63] Bari A., Robbins T.W. (2013). Inhibition and impulsivity: Behavioral and neural basis of response control. Prog. Neurobiol..

[64] Aron A.R., Cai W., Badre D., Robbins T.W. (2015). Evidence supports specific braking function for inferior PFC. Trends Cogn. Sci..

[65] West R. (2003). Neural correlates of cognitive control and conflict detection in the Stroop and digit-location tasks. Neuropsychologia.

[66] West R., Jakubek K., Wymbs N., Perry M., Moore K. (2005). Neural correlates of conflict processing. Exp. Brain Res..

[67] Chmielewski W.X., Tiedt A., Bluschke A., Dippel G., Roessner V., Beste C. (2018). Effects of multisensory stimuli on inhibitory control in adolescent ADHD: It is the content of information that matters. NeuroImage Clin..

[68] Fallgatter A.J., Ehlis A.-C., Rösler M., Strik W.K., Blocher D., Herrmann M.J. (2005). Diminished prefrontal brain function in adults with psychopathology in childhood related to attention deficit hyperactivity disorder. Psychiatry Res..

[69] Fallgatter A.J., Ehlis A.-C., Seifert J., Strik W.K., Scheuerpflug P., Zillessen K.E., Herrmann M.J., Warnke A. (2004). Altered response control and anterior cingulate function in attention-deficit/hyperactivity disorder boys. Clin. Neurophysiol. Off. J. Int. Fed. Clin. Neurophysiol..

[70] Paul-Jordanov I., Bechtold M., Gawrilow C. (2010). Methylphenidate and if-then plans are comparable in modulating the P300 and increasing response inhibition in children with ADHD. Atten. Deficit Hyperact. Disord..

[71] Pliszka S.R., Liotti M., Bailey B.Y., Perez R., Glahn D., Semrud-Clikeman M. (2007). Electrophysiological effects of stimulant treatment on inhibitory control in children with attention-deficit/hyperactivity disorder. J. Child Adolesc. Psychopharmacol..

[72] Seifert J., Scheuerpflug P., Zillessen K.-E., Fallgatter A., Warnke A. (2003). Electrophysiological investigation of the effectiveness of methylphenidate in children with and without ADHD. J. Neural Transm..

[73] Van der Oord S., Geurts H.M., Prins P.J.M., Emmelkamp P.M.G., Oosterlaan J. (2012). Prepotent response inhibition predicts treatment outcome in attention deficit/hyperactivity disorder. Child Neuropsychol. J. Norm. Abnorm. Dev. Child. Adolesc..

[74] Bluschke A., Broschwitz F., Kohl S., Roessner V., Beste C. (2016). The neuronal mechanisms underlying improvement of impulsivity in ADHD by theta/beta neurofeedback. Sci. Rep..

[75] Bluschke A., Roessner V., Beste C. (2016). Specific cognitive-neurophysiological processes predict impulsivity in the childhood attention-deficit/hyperactivity disorder combined subtype. Psychol. Med..

[76] Navarra R., Graf R., Huang Y., Logue S., Comery T., Hughes Z., Day M. (2008). Effects of atomoxetine and methylphenidate on attention and impulsivity in the 5-choice serial reaction time test. Prog. Neuropsychopharmacol. Biol. Psychiatry.

[77] Bedard A.-C.V., Stein M.A., Halperin J.M., Krone B., Rajwan E., Newcorn J.H. (2015). Diffenrtial impact of methylphenidate and atomoxetine on sustained attention in youth with attention-deficit/hyperactivity disorder. J. Child. Psychol. Psychiatry.

[78] Nakanishi Y., Ota T., Iida J., Yamamuro K., Kishimotor N., Okzaki K., Kishimoto T. (2017). Differential therapeutic effects of atomoxetine and methylphenidate in childhood attention deficit/hyperactivity disorder as measured by near-infrared spectroscopy. Child. Adolesc. Psychiatry Ment. Health.

[79] Schulz K.P., Fan J., Bedard A.C.V., Clerkin S.M., Ivanov I., Tang C., Halperin J., Newcorn J.H. (2012). Common and unique therapeutic mechanisms of stimulant and nonstimulant treatments for attention-deficit/hyperactivity disorder. Arch. Gen. Psychiatry.

[80] Hermens D.F., Rowe D.L., Gordon E., Williams L.M. (2006). Integrative neuroscience approach to predict ADHD stimulant response. Expert Rev. Neurother..

[81] Stein M.A., MCGough J.J. (2008). The pharmacogenomic era: Promise for personalizing ADHD therapy. Child. Adolesc. Psychiatr. Clin. N. Am..

